# Retraction: Sirtuin1 role in the melatonin protective effects against obesity-related heart injury

**DOI:** 10.3389/fphys.2023.1328892

**Published:** 2023-11-06

**Authors:** Frontiers Editorial Office

The journal retracts the 2020 article cited above.

Following publication, concerns were raised regarding the integrity of the images in Figure 2A as published. Specifically, the images presented in Figure 2A were found to have been previously published by a different research group as Figure 5(a), in the Journal of Nutrition and Metabolism (2017), DOI:10.1155/2017/4964571.

The authors failed to provide the raw data pertaining to the figure in question nor a satisfactory explanation during the investigation, which was conducted in accordance with Frontiers’ policies. As a result, the data and conclusions of the article have been deemed unreliable and the article has been retracted.

The authors have not responded to correspondence regarding this retraction.

